# ChlVPP combination chemotherapy for Hodgkin's disease: long-term results.

**DOI:** 10.1038/bjc.1990.278

**Published:** 1990-08

**Authors:** P. Selby, P. Patel, S. Milan, M. Meldrum, J. Mansi, E. Mbidde, M. Brada, T. Perren, G. Forgeson, M. Gore

**Affiliations:** Section of Medicine, Institute of Cancer Research, Royal Marsden Hospital, Sutton, Surrey, UK.

## Abstract

Two hundred and eighty-four patients with advanced Hodgkin's disease (HD) (stage II with poor prognostic features and stage III/IV) have been treated with the ChlVPP combination chemotherapy regimen (chlorambucil, vinblastine, procarbazine and prednisolone) in a single-centre unselected series. Median follow up is 92 months. Fifty-five patients had previously received radiotherapy but none had received previous chemotherapy. Eighty-five per cent of previously untreated patients and 91% of previously irradiated patients entered complete remission (CR); 71% and 68% of these respectively remain in CR at 10 years and 65% and 64% of each group respectively are alive at 10 years. On univariate analysis, age, stage, site of visceral disease and lymphocyte count predicted survival and on multivariate analysis age, absence of symptoms, absence of lung, liver or bone marrow disease and achieving a CR remained important predictors of survival. Acute toxicity was mild. The 10 year actuarial risk of acute leukaemia was 2.7%. This study adds further support to the view that chlorambucil is as effective and less toxic than mustine in combination chemotherapy for HD. We suggest that MOPP chemotherapy is no longer routinely indicated for HD.


Br. J. Cancer (1990), 62, 279-285 © Macmillan Press Ltd., 1990

### ChlVPP combination chemotherapy for Hodgkin's disease: long term results

P. Selby, P. Patel, S. Milan[1], M. Meldrum, J. Mansi, E. Mbidde, M. Brada, T. Perren, G. Forgeson, M. Gore, I. Smith & T. McElwain

*Section of Medicine and [1]Department of Computing, Institute of Cancer Research, Royal Marsden Hospital, Sutton, Surrey, UK.*

**Summary** Two hundred and eighty-four patients with advanced Hodgkin's disease (HD) (stage II with poor prognostic features and stage III/IV) have been treated with the ChlVPP combination chemotherapy regimen (chlorambucil, vinblastine, procarbazine and prednisolone) in a single-centre unselected series. Median follow up is 92 months. Fifty-five patients had previously received radiotherapy but none had received previous chemotherapy. Eighty-five per cent of previously untreated patients and 91% of previously irradiated patients entered complete remission (CR); 71% and 68% of these respectively remain in CR at 10 years and 65% and 64% of each group respectively are alive at 10 years. On univariate analysis, age, stage, site of visceral disease and lymphocyte count predicted survival and on multivariate analysis age, absence of symptoms, absence of lung, liver or bone marrow disease and achieving a CR remained important predictors of survival. Acute toxicity was mild. The 10 year actuarial risk of acute leukaemia was 2.7%. This study adds further support to the view that chlorambucil is as effective and less toxic than mustine in combination chemotherapy for HD. We suggest that MOPP chemotherapy is no longer routinely indicated for HD.

The successful introduction of the MOPP combination chemotherapy regimen in 1964 at the National Cancer Institute (US) has proved to be a huge step forward in the treatment of Hodgkin's disease (De Vita *et al.*, 1980; Longo *et al.*, 1986). The complete remission rate of 84% with overall survival of 48% for a group of patients, most of whom had advanced disease, remains the standard by which alternative treatments for Hodgkin's disease must be measured. Nevertheless, there are substantial residual problems with the chemotherapy of Hodgkin's disease. Its efficacy is still limited and 40-50% of patients either fail to enter complete remission or relapse after chemotherapy with MOPP. The toxicity of MOPP and related combinations is substantial with acute haematological, gastroenterological and neurological toxicity followed by long term gonadal toxicity and the development of second malignancies, particularly acute myeloid leukaemia (Selby *et al.*, 1987). For the past 20 years, research on the chemotherapy of Hodgkin's disease has sought to reduce the toxicity and increase the efficacy of chemotherapy regimens.

In 1975 we introduced the ChlVPP (chlorambucil, vinblastine, procarbazine and prednisolone) regimen at the Royal Marsden Hospital. In our practice this superseded a MOPP-based regimen known as MVPP in which vincristine was replaced with vinblastine. Analyses in 1977 and 1982 suggested that the ChlVPP regimen was an effective low-toxicity combination chemotherapy for Hodgkin's disease (McElwain *et al.*, 1977; Dady *et al.*, 1982). Moreover, a randomised prospective trial compared chlorambucil-based combination chemotherapy with mustine-based combination chemotherapy and concluded that the regimens were of comparable efficacy (British National Lymphoma Investigation, 1986). In this report, we describe the long term efficacy and toxicity of the ChlVPP regimen in 284 adults who had received no previous chemotherapy for their Hodgkin's disease.

### Patients and methods

Between January 1975 and March 1986, 284 patients were treated with ChlVPP combination chemotherapy including 55 who had received previous radiotherapy. No patients had received previous chemotherapy. Follow-up was available on 274 patients to January 1989. Ten patients have follow-up for less than two years having left the UK and these are censored in the analysis at the point of last visit to RMH. All patients were seen and treated at the Royal Marsden Hospital, Sutton and represent an unselected series of patients referred to that hospital. During the period of this study between November 1981 and January 1983 patients were entered into a separate study in which procarbazine was replaced by etoposide in the regimen (the OPEC regimen). During the period of the OPEC study all patients were entered in an unselected way and no excluded patients received the ChlVPP regimen alone. From March 1986 ChlVPP ceased to be the uniform first choice chemotherapy at the hospital. Patients who received ChlVPP after March 1986 were selected according to patient or physician preference and are not included in this series. Children of age less than 16 years were excluded and are the subject of a previous report (Robinson *et al.*, 1984).

All records were reviewed retrospectively and for each reviewer at least 10% of the findings were independently checked by one other reviewer. If more than 5% of the findings of a single contributor were in disagreement with the second reviewer, a complete reanalysis of those patients was performed. Bulk of disease at peripheral sites was defined as masses greater than 5 cm and bulk in the mediastinum was defined as a ratio of the maximum diameter of the mass to the maximum diameter of the thorax of greater than 33%. Haematological and biochemical tests were recorded and the records of all imaging tests were reviewed. Histology was reviewed for all patients at RMH. Histological classification was by the criteria of Lukes and Butler (1966), and staging was according to the Ann Arbor System (Carbone *et al.*, 1971). Toxicity according to WHO scales (World Health Organization, 1979) was retrospectively analysed by review of the hospital records.

Patients were assessed by a full clinical history and physical examination, full blood count, erythrocyte sedimentation rate, serum biochemistry and liver function tests, chest X-ray, lymphogram and bone marrow aspirates with trephine. CT scan of the mediastinum and abdomen, hepatic ultrasound and isotope liver scan, gallium scan and staging laparotomy were performed only when clinically indicated. A staging laparotomy was performed in 104 of 229 previously untreated patients and 6 of 55 who had had previous radiotherapy. During treatment, patients were reassessed prior to each course of ChlVPP by physical examination, chest X-ray, abdominal X-ray, and full blood count. At the end of chemotherapy patients were restaged by repeating all

Correspondence: P. Selby, Institute for Cancer Studies, St James's University Hospital, Beckett Street, Leeds LS9 7TF, UK.
Received 6 October 1989; and in revised form 28 February 1990.

investigations which had been found to be abnormal before chemotherapy was given. The timing of complete remission is therefore defined by physical examination and the interim investigations but the proportion of patients entering complete remission is defined by full restaging investigations. A complete remission is defined as a return to normal for all findings on examination and on repeat of all previously abnormal investigations. The significance of an abnormal mediastinal contour on chest X-ray may present great difficulty in interpretation and is discussed below.

For patients who have relapsed from previous radiotherapy, the Ann Arbor Staging System should not be applied directly and it was not designed for this purpose (Carbone et al., 1971). In attributing stages to such patients, we have used the Ann Arbor recommendations but included all known sites of disease (before and after radiotherapy) in the estimation of stage. These allocations of 'cumulative stage' at relapse must be distinguished from stage at presentation.

### *Selection of patients for chemotherapy and radiotherapy*

The indications for chemotherapy employed at RMH in the period 1975–1986 were consistent. However, the addition of radiotherapy was not defined by the stage. The addition of radiotherapy to sites of bulky disease was usual but not invariable in patients with stage III or IV disease and the use of extended field radiotherapy after chemotherapy in stage I and II disease was usual but not invariable. All patients with stage IV disease who had not had previous radiotherapy received chemotherapy and radiotherapy was added to sites of bulky disease in 8 cases. All patients with clinical stage III disease and patients with pathological stage III with splenic disease received chemotherapy first and this was followed by radiotherapy in 28 of 83 cases. Patients with stage I and II disease of poor prognosis (B symptoms, bulky disease, >3 nodal sites, 'E' extra nodal extension) received chemotherapy first and this was followed by radiotherapy in 65 of 84 cases.

### *Treatment regimen*

Days 1–14 inclusive: chlorambucil 6 mg m$^{-2}$ per day orally (not exceeding 10 mg per day); procarbazine 100 mg m$^{-2}$ per day orally (not exceeding 150 mg per day); prednisolone 40 mg per day orally. Days 1 and 8: vinblastine 6 mg m$^{-2}$ intravenously (not exceeding 10 mg per single dose).

Treatment was repeated every 4 weeks with one week's delay if the leucocyte count was less than $3 \times 10^9\,l^{-1}$, the neutrophil count was less than $1.5 \times 10^9\,l^{-1}$ or platelets were less than $100 \times 10^9\,l^{-1}$. Vinblastine was reduced to 3 mg m$^{-2}$ if grade II neuropathy developed. The number of treatment cycles was judged according to the response of the patient. Patients were treated to complete remission plus at least two further cycles. In the first few years of the study, more prolonged treatment beyond remission was employed in 38 patients (see below).

### *Radiotherapy*

Radiotherapy started 6 weeks after the last course of chemotherapy. The extended field (mantle, inverted Y or total nodal) appropriate to the extent of the disease at presentation was treated with 35 Gy mid plain dose in 20 fractions per 4 week course. Fifty-three patients received mantle radiotherapy, 13 patients mantle plus para aortic strip, eight patients inverted Y and 39 patients total nodal irradiation. Fifteen patients received modified field radiotherapy to the same dose.

### *Statistical methods*

Differences in patient characteristics and comparisons of complete remission rates were evaluated using the $\chi^2$ test with Yates' correction for categorical variables, or the Mann–Whitney rank test where one factor is continuous.

Survival duration and relapse-free interval for patients who achieved complete remission were both measured from the date of first chemotherapy. The curves presented were calculated using the method of Kaplan and Meier (1978) and the log rank test (Peto et al., 1977) was used to compare the curves. The percentages of patients remaining in remission and surviving at 5 and 10 years given in Tables I and II are obtained from such curves. For continuous variables, different groupings were examined to determine those which most influenced survival and remission durations. A stepwise linear regression analysis based on Cox's proportional hazards model (Cox, 1972) was performed to assess the relative importance of the various factors in determining the survival duration and relapse-free interval for this group of patients.

### Results

### *Patient characteristics*

Table I includes the basic demographic and clinical features of the 229 patients who had had no previous treatment. In Table II abbreviated data are given for 55 patients who had relapsed from previous radiotherapy. The median follow-up for surviving patients is 92 months.

### *Treatment*

The median number of courses for all patients was 6 (range 1–16) and 174 of 284 patients received 6 courses. Seven patients received 7 courses; 14 received 8 courses; 4 received 9 courses; 12 received 10 courses and 1 received 16 courses. One hundred and twenty-eight previously untreated patients received elective radiotherapy following their chemotherapy.

### *Efficacy*

Tables I and II give complete remission rates together with the observed probability of continuing remission and survival at 5 and 10 years after treatment. The overall CR rate was 85% (no previous treatment) and 91% (previous radiotherapy). The 10 years probability of survival was 65% and 67% for these two groups respectively and 20–25% of CR patients relapsed. Duration of remission and survival curves are shown in Figures 1 and 2. The overall survival and relapse curves for patients who had no previous treatment and those who had previous radiotherapy are similar (Figure 1a and b).

Among 128 patients who received radiotherapy after chemotherapy the CR rate was 95% while among 101 patients who received chemotherapy alone the CR rate was 71%. However, the policy in patients who failed to remit on ChlVPP chemotherapy was to switch to alternative chemotherapy regimens, which means that the selection of cases for the no-radiotherapy group was influenced by their response and the difference between the two groups is inevitably biased. There were 21 patients within the group who received elective radiotherapy after chemotherapy who entered CR after receiving combined modality treatment but were not felt to be in CR after the chemotherapy. Fourteen of these had residual abnormalities of the mediastinal contour after shrinkage of bulky mediastinal disease and the true significance of this remains uncertain. The remaining 7 had residual disease at other sites after chemotherapy.

For previously untreated patients Table III summarises the results of a univariate analysis to examine the influence of all of the factors listed in Table I as well as ESR, haemoglobin, alkaline phosphatase, hepatic transaminase, gamma glutamyl transpeptidase on CR, relapse and survival. The important prognostic factors which emerged from this analysis for the previously untreated group of patients are given below.

*Age* (Figure 2a and b). The age distribution is unremarkable for HD with median 30, range 16–81. The main prog-

**Table I** 229 no previous treatment ChlVPP patients

| | | No. patients | No. CR (%) | Remaining in remission 5 years % | Remaining in remission 10 years % | Survival 5 years % | Survival 10 years % |
|---|---|---|---|---|---|---|---|
| Total | | 229 | 194 (85%) | 74.4 | 71.3 | 73.1 | 65.2 |
| Sex | Male | 152 | 126 (83%) | 72.1 | 67.5 | 84.1 | 73.5 |
| | Female | 77 | 68 (88%) | 78.7 | 78.7 | 83.9 | 79.5 |
| Histology | LD | 10 | 8 (80%) | 75.0 | 37.5 | 70.0 | 46.7 |
| | LP | 12 | 9 (75%) | 41.7 | 41.7 | 65.6 | 52.5 |
| | MC | 70 | 62 (89%) | 83.2 | 80.5 | 78.2 | 67.5 |
| | NS | 137 | 115 (84%) | 72.1 | 70.3 | 71.3 | 65.8 |
| Clinical Stage | I | 27 | 26 (96%) | 75.2 | 65.8 | 79.3 | 70.5 |
| | II | 89 | 78 (88%) | 79.4 | 76.2 | 79.9 | 77.8 |
| | III | 64 | 55 (86%) | 68.6 | 66.0 | 71.3 | 58.2 |
| | IV | 49 | 35 (71%) | 71.3 | 71.3 | 59.4 | 51.6 |
| Final Stage (after LAP) | I | 13 | 12 (92%) | 90.0 | 60.0 | 80.0 | 53.3 |
| | II | 71 | 62 (87%) | 77.3 | 77.3 | 76.8 | 72.5 |
| | III | 83 | 74 (77%) | 75.3 | 70.9 | 74.5 | 70.7 |
| | IV | 62 | 46 (74%) | 64.3 | 64.3 | 64.8 | 50.9 |
| B Symptoms | No | 117 | 107 (91%) | 76.4 | 72.0 | 80.5 | 73.5 |
| | Yes | 112 | 87 (78%) | 71.7 | 69.9 | 65.3 | 56.5 |
| Age | <26 | 74 | 66 (89%) | 82.4 | 79.4 | 86.0 | 86.0 |
| | 26–39 | 88 | 71 (81%) | 74.3 | 72.3 | 74.6 | 68.0 |
| | 40–59 | 43 | 38 (88%) | 61.5 | 61.5 | 66.3 | 39.6 |
| | 60+ | 24 | 19 (79%) | 65.7 | 52.6 | 39.7 | 21.7 |
| Lung | No | 197 | 171 (87%) | 75.0 | 71.7 | 75.2 | 66.2 |
| | Yes | 16 | 10 (62%) | 66.7 | – | 53.6 | 53.6 |
| | Early | 16 | 13 (81%) | 76.9 | 76.9 | 72.2 | 72.2 |
| Liver | No | 193 | 166 (86%) | 78.3 | 74.6 | 74.4 | 70.4 |
| | Yes | 36 | 28 (78%) | 55.9 | 55.9 | 69.0 | 48.2 |
| Bone marrow | No | 220 | 190 (86%) | 74.7 | 71.6 | 74.8 | 66.5 |
| | Yes | 9 | 4 (44%) | 75.0 | 75.0 | 40.0 | 40.0 |
| Lung, Liver, bone marrow | Yes | 53 | 38 (72%) | 57.5 | 57.4 | 62.6 | 46.9 |
| | No | 176 | 156 (89%) | 78.6 | 74.7 | 76.4 | 72.0 |
| Bone | No | 224 | 190 (85%) | 75.2 | 72.1 | 74.1 | 66.0 |
| | Yes | 5 | 3 (60%) | 66.7 | 66.7 | 40.0 | 40.0 |
| Pleural effusion | No | 207 | 179 (85%) | 73.8 | 70.4 | 74.1 | 66.2 |
| | Yes | 22 | 15 (68%) | 85.7 | 85.7 | 67.0 | 60.3 |
| Spleen | No | 139 | 116 (83%) | 76.2 | 72.4 | 71.8 | 65.6 |
| | Yes | 90 | 78 (87%) | 72.7 | 70.6 | 75.8 | 66.5 |
| Mediastinum | No | 117 | 99 (84%) | 74.0 | 68.4 | 72.1 | 64.0 |
| | Yes | 111 | 95 (85%) | 75.5 | 75.5 | 75.0 | 66.8 |
| Bulky mediastinum | No | 51 | 45 (88%) | 78.8 | 78.8 | 78.6 | 65.1 |
| | Yes | 58 | 48 (82%) | 75.8 | 75.8 | 72.4 | 68.6 |
| No of nodal sites | 0–3 | 126 | 108 (86%) | 78.5 | 76.3 | 74.8 | 71.6 |
| | >3 | 103 | 86 (84%) | 69.0 | 65.2 | 72.4 | 59.1 |
| Bulky nodes | No | 106 | 92 (87%) | 71.4 | 69.0 | 72.2 | 64.1 |
| | 1 | 103 | 86 (83%) | 74.3 | 69.7 | 71.1 | 67.1 |
| | >1 | 20 | 16 (80%) | 93.8 | 93.8 | 90.0 | 69.8 |
| Response | CR | 194 | – | – | – | 84.0 | 75.8 |
| | Non CR | 35 | – | – | – | 14.8 | 9.9 |

**Table II** Patients relapsing from previous radiotherapy

| | | No. of pts | CR (%) | Remission 5 years % | Remission 10 years % | Survival 5 years % | Survival 10 years % |
|---|---|---|---|---|---|---|---|
| Total | | 55 | 50 (91%) | 80.7 | 76.4 | 71.3 | 66.7 |
| Stage[a] | I | 7 | 7 | 100 | 100 | 100 | 100 |
| | II | 8 | 5 | 50 | 50 | 42.9 | 42.9 |
| | III | 24 | 22 | 95 | 87.7 | 78.0 | 73.1 |
| | IV | 16 | 16 | 61.1 | 61.1 | 60.9 | 50.8 |
| | A | 42 | 38 | 85.5 | 80.2 | 74.3 | 68.1 |
| | B | 13 | 12 | 66.7 | 66.7 | 61.5 | 61.5 |
| Age | <26 | 12 | 11 | 90.0 | 75.0 | 82.5 | 73.3 |
| | 26–39 | 29 | 26 | 71.8 | 71.8 | 74.7 | 74.7 |
| | 40–59 | 11 | 10 | 88.9 | 88.9 | 63.6 | 50.9 |
| | >59 | 3 | 3 | – | – | – | – |

[a]Stage attributed by adding all sites of disease including presentation and first relapse.

**Figure 1** a, Actuarial probability of remaining in remission for those patients who entered CR after ChlVPP who had no previous treatment (NPT: 194 patients) or who had previous radiotherapy (RT: 50 patients). Vertical marks indicate censored patients. b, Actuarial probability of survival after ChlVPP chemotherapy for patients who had no previous treatment (229 patients) or had previous radiotherapy (55 patients). Vertical marks indicate censored patients.

**Table III** Significance levels of prognostic factors on univariate analysis for previously untreated patients

| | *CR* | *5 years relapse from CR* | *Survival* |
|---|---|---|---|
| Age | NS | 0.039 | >0.001 |
| Stage <III v IV (IV worse) | 0.013 | NS | 0.007 |
| A v B (B worse) | 0.007 | NS | 0.001 |
| Lung (worse) | 0.028 | NS | 0.034 |
| Marrow (worse) | 0.003 | NS | 0.084 |
| Liver | NS | 0.039 | 0.035 |
| Number of nodal sites <3 v 3 | NS | 0.076 | NS |
| Achieving CR | – | – | <0.001 |
| Lymphocytes $<1\times10^9/l$ | 0.028 | NS | 0.018 |

Sex, bone disease, histology, pleural effusion, ESR, haemoglobin, gamma GT, transaminase were not sigificant at the 5% level in this analysis.

nostic influence of age lies in the increased risk of death in remission for those over 40 years (3 of 174 under 40 years *vs* 14 of 70 over 40 years) and 7 of the deaths in CR over 40 years of age were early (less than 1 year following the start of ChlVPP). Five of these were due to infection. The increased risk of death in remission is significant ($P<0.00001$) and together with a marginally higher relapse risk ($P=0.039$) results in a highly significant reduction in survival for older patients.

*Sex* The series contains more men (55%) than women (44%) but there is no significant difference in CR, relapse, survival or death in remission.

*Histology* The expected excess of nodular sclerosis subtype (60%) over mixed cellularity (31%) and lymphocyte predominant (5%) or depleted (4%) is seen and NS patients are younger (median 29 years *vs* median 35 years for MC, $P=0.0085$). Most NS cases were classified as mixed cellular

**Figure 2** a, Actuarial probability of remaining in remission after ChlVPP chemotherapy for those patients who entered CR divided according to age. The curves are labelled with age grouping in years. No previous therapy. b, Actuarial probability of survival after ChlVPP chemotherapy divided according to age. The curves are labelled with age groupings in years. No previous therapy. c, Actuarial probability of remaining in remission after CR. A *vs* B stage. No previous therapy. d, Actuarial probability of survival. A *vs* B stage. No previous therapy. e, Actuarial probability of remaining in remission after CR. Stage I and II (solid line), II (dashed line), IV (dot-dash). No previous therapy. f, Actuarial probability of survival. Stage I and II (solid line), II (dashed line), IV (dot-dash). No previous therapy.

content for the nodules (77%). Overall the histological subtype did not influence outcome. Although the relapse rate in the small number of LD patients was high (62.5% relapsed at 10 years) this did not achieve significance.

*Stage* The significance of stage in this series is likely to be diluted since lower stage cases were selected for chemotherapy by their poor prognostic features. If stage I–III patients are compared to stage IV then a highly significant effect on survival is seen ($P < 0.007$) which results from lower CR rates ($P = 0.013$), and somewhat more relapses and deaths in remission (not significant). Patients with B symptoms have worse survival due mainly to a lower CR rate (Figure 2c–f).

*Sites of disease* The presence of lung, liver or marrow disease as well as having more than three involved nodal sites predicted lower survival compared to patients without any of these factors. For lung and marrow disease this was due to lower CR rate and more relapse whereas for liver disease only the relapse rate was significantly higher at $P < 0.05$.

*Lymphocyte count* An absolute lymphocyte count of less than $1 \times 10^9 l^{-1}$ was associated with a lower CR rate and survival.

The effect of previous radiotherapy was then analysed by univariate analysis of the whole patient group (284 patients). Previous radiotherapy was not a significant factor predicting response, relapse or survival. The number of courses of ChlVPP required to achieve CR varied between 1 and 9 with a median of 3 courses. The number of courses required to achieve CR was not a significant predictor of relapse or survival.

### *Multivariate analysis*

The multivariate analysis was performed in 284 patients and included all the variables listed for univariate analysis except for lymphocyte count (data inadequate).

The factors which independently predicted for improved survival were achieving a complete remission, younger age, absence of symptoms, and absence of lung, liver and marrow disease. The individual visceral sites of disease were not significant factors. If remission status was removed from the analysis then the same other factors remained significant.

Among patients who entered CR, relapse was less likely in patients who had no lung, liver or marrow disease and in those who had less than 3 nodal sites. Among the CR patients, factors predicting survival were age and sites of disease reflecting the importance of age in predicting death in remission.

### *Toxicity*

The toxicity of the regimen was recorded retrospectively for each course in each patient. This is summarised in Table IV for the maximum recorded toxicity on any course in any patient. Acute toxicity was very modest. Moderate degrees of myelosuppression occurred but in a minority of patients; nausea and vomiting were mild and uncommon; alopecia or moderate neuropathy were rare. Two patients died of infection during their ChlVPP chemotherapy. They were not leucopenic.

**Table IV** World Health Organization graded toxicity

| | % patients | | | |
|---|---|---|---|---|
| | *I* | *II* | *III* | *IV* |
| Anaemia | 20 | 9 | 0 | 0 |
| Leukopenia | 21 | 20 | 7 | 2 |
| Thrombocytopenia | 8 | 10 | 4 | 0.5 |
| Nausea and Vomiting | 18 | 13 | 1.5 | 0.5 |
| Alopecia | 5 | 1.5 | 0.5 | 0 |
| Neuropathy | 11 | 3 | 0 | 0 |
| Infection | 8 | 9 | 1.5 | 1.5 |
| Diarrhoea | 2.5 | 0.5 | 0 | 0 |
| Stomatitis | 1.5 | 1 | 0 | 0 |

### *Second malignancies*

With median follow up of 92 months, we can now comment on the rate of second malignancy. Two cases of acute myeloid leukaemia were seen at 7.5 and 8.7 years in patients who had received 9 and 16 courses of ChlVPP respectively without radiotherapy and without relapse. The actuarial risk of a secondary leukaemia is 2.7% at 10 years. Twelve other second cancers were noted: one malignant melanoma, one stomach cancer, two carcinoma of bronchus, one non-Hodgkin's lymphoma, one breast cancer, one pancreatic cancer and five basal cell carcinomas. The actuarial risk of any second malignancy is 8.3% at 10 years.

### *Dose reduction and delay*

Details of exact dosage of drug prescribed in each course was available for 209 patients of the 229 who had received no previous treatment. The number with *any* reduction for each course is shown in Table V together with the proportion of cases for whom treatment was delayed. These represent a very small proportion of cases and the prognostic significance of reduction and delay in dosage has not been analysed.

### *Cause of death*

Eighty-eight patients have died in the whole series (284 patients) of whom 38 were considered to have died of progressive Hodgkin's disease only. Twenty-nine more patients had active Hodgkin's disease when they died but among these 3 died of a second malignancy, one myocardial infarction, one pulmonary embolus, two of complications of autologous bone marrow transplantation as salvage therapy and 22 of infection. Among these 22 patients, 20 had relapsed at least once while two patients died of intercurrent infection during their first course of ChlVPP.

Sixteen patients died during complete remissions of their Hodgkin's disease. Seven died of infection within one year of their chemotherapy and these deaths may be attributed to disease and treatment related immunosuppression. One died of late complications of autologous bone marrow transplantation. Eight patients died of miscellaneous causes which were not apparently linked to Hodgkin's disease or treatment.

Figure 3 is a partial cumulative probability plot relating survival to cause of death. The majority of patients died of or with active Hodgkin's disease and among those who died in remission, immunosuppression was the most important factor. Second malignancies – only some of which may be linked to treatment – were only a minor cause of death in this series (Figure 3).

### Discussion

The results described for the efficacy of the ChlVPP regimen in this single institution series compare favourably with any previous results described for the combination chemotherapy of advanced Hodgkin's disease with MOPP or regimens

**Table V** Dose reduction or delay

| | Course | | | | | |
|---|---|---|---|---|---|---|
| | *1* | *2* | *3* | *4* | *5* | *6* |
| % of pts with any dosage reduction | 6 | 13 | 13 | 12 | 16 | 15 |
| % of pts with 1 week delay | – | 4 | 7 | 4 | 11 | 5 |
| % of pts with 2 weeks delay | – | 6 | 4 | 6 | 7 | 10 |

**Figure 3** Actuarial probability of survival according to cause of death. Curve (**a**) shows deaths due to active HD and all other deaths are censored. In curve (**b**) deaths due to second malignancy occurring patients with active HD are added to those in curve (**a**) showing the increment in deaths due to this cause. In curve (**c**) the deaths in CR excluding those due to second malignancies are added to those shown in curve (**b**). In curve (**d**) all deaths are shown, those due to second malignancy in CR are added to those shown in curve (**c**). The difference between (**c**) and (**d**) may be taken to indicate the difference in outcome for HD patients resulting from all second cancers only some of which are due to the carcinogenic effect of ChlVPP.

derived from it. In the present study, the complete remission rate was 85% among patients who had had no previous treatment with a 5-year probability of remaining in complete remission of 74%. In a recent review of the outcome of treatment with MOPP and its variants, the range of complete remissions reported was from 62 to 82% with actuarial 4-year relapse-free survival of between 50 and 80% of these patients who achieved CR (Selby *et al.*, 1987). Our results are in keeping with long term outcomes reported for 54 patients treated with ChlVPP in Southampton (McKendrick *et al.*, 1989). In a recent update of the 198 patients originally treated with MOPP in the classic series from the National Cancer Institute (US) the revised complete remission rate was 84% with 66% patients remaining relapse free (Longo *et al.*, 1986).

We emphasise that comparisons between series in different insititutions must of course be interpreted very cautiously because of differing patient populations accumulated into multi institutional or single institutional studies of this kind. For instance, over 50% of our patients were free of B symptoms whereas only 12% of NCI patients were symptom-free. It is not possible to compare the series by examining published reports in a conclusive way. Even a meta-analysis of results in a single database may not be sufficient. However, a randomised prospective trial in which mustine-containing combination chemotherapy (MOPP) and chlorambucil-containing chemotherapy (LOPP) were compared showed no advantage to the use of the mustine based treatment (British National Lymphoma Investigation, 1986). This trial supports the equivalent efficacy of mustine and chlorambucil. Even though the overall results were poor, they were similar in each arm of the trial. The literature contains at least four trials in which mustine-containing regimens have been compared to regimens containing other alkylating agents (Bakemeier *et al.*, 1984; reviewed by Selby *et al.*, 1987). In no case was mustine found to be more effective than the alternatives. We feel that all of the available data support our view that alternative alkylating agents, including chlorambucil, are as effective as mustine in combination chemotherapy for Hodgkin's disease.

The acute toxicity described in this paper is substantially less than the reported acute toxicity of MOPP (for review see Selby *et al.*, 1987). Myelosuppression, nausea and vomiting, neuropathy and alopecia are substantially lower with chlorambucil based combination chemotherapy and the need for venous access for the administration of mustine and the complexities of the rapid administration of mustine before chemical degradation occur are avoided with ChlVPP. We are reassured that the long term observations in this study suggest that chlorambucil based combination chemotherapy does not appear to be more leukaemogenic or carcinogenic than mustine-based combination chemotherapy for Hodgkin's disease. The observed 10-year actuarial risk of acute leukaemia in this series was 2.7%, both cases occurring in patients who had chemotherapy without radiotherapy. The literature contains figures for 10 year leukaemia risks of 1.2–15.6% for all patients who have chemotherapy with MOPP or its variants (Tucker *et al.*, 1988; Colman & Selby, 1987).

The use of mustine based combination chemotherapy is now inappropriate in the management of Hodgkin's disease and owes more to tradition than to clinical science. There are equally effective alternative alkylating agents and among these, chlorambucil is one less toxic choice.

The ChlVPP regimen can be given in full doses and without delay to the majority of patients which may be in part responsible for the observed efficacy. The small number of patients for whom dose reductions or delays are necessary precludes a formal analysis of the intensity of treatment as a determinant of outcome in this study. The rather lower complete remission and survival rates observed with other chlorambucil based regimens which have lower doses of chlorambucil and procarbazine (British National Lymphoma Investigation, 1986) suggest that the treatment should be used in the doses described in this paper without dose reduction or modification if this can be avoided.

The factors which we have observed to influence the outcome of treatment with the ChlVPP regimen are broadly in keeping with those seen in other series with other regimens (see Selby *et al.*, 1987). Age and disease affecting liver, lung and bone marrow are powerful predictors of outcome and achieving CR is the most important determinant of survival. Some differences exist between the importance of the factors described here and important prognostic factors found elsewhere in the literature (Wagstaff *et al.*, 1988; reviewed by Selby *et al.*, 1987). These differences are more likely to be due to differing patient populations and selection together with differing institutional practice for investigation and data collection than to be due to any real biological differences. For instance, Wagstaff *et al.* (1988) found age, sex, lymphocyte count and stage to be independent indicators of survival in the Barts/Christie series. We were unable to include lymphocyte count in our multivariate analysis because our data were incomplete. We found no difference in survival according to sex on univariate or multivariate analysis. We cannot fully explain the discrepancy but the patient populations differ in stage and treatment so that all comparisons are tentative. For these reasons we do not propose to develop any prognostic models or indices based upon the observations in the present series.

The development of ChlVPP combination chemotherapy has reduced the toxicity and need for hospitalisation of patients undergoing chemotherapy for Hodgkin's disease. It can be given easily, routinely and safely in outpatients and many patients continue to work normally. This regimen is, however, associated with secondary acute leukaemia in a small number of cases in this series and is associated with infertility in the majority of men and a minority of younger women (Sutcliffe, 1987).

The principal problem in the future development of chemotherapy for Hodgkin's disease remains the need for more effective treatment which will reduce the 30–50% of patients whose disease is not cured by existing regimens. Failure to remit and early relapse are the major risks to our patients. The advantages of adriamycin based regimens or of alternation of alkylating agent based regimens with adriamycin based regimens remain uncertain but recent clinical trials are suggesting an advantage for the use of an adriamycin based regimen such as ABVD either alone or in alternation with MOPP or its variants (Bonnadonna *et al.*, 1985; review by Selby *et al.*, 1987). In addition the use of high dose treatment with autologous bone marrow grafting is allowing salvage of a proportion of patients who relapse from conventional dosage chemotherapy (Russell *et al.*, 1989; Zulian *et al.*, 1989). Chlorambucil based combination chemotherapy represents a substantial step towards reducing the burden of combination chemotherapy upon the patient with advanced Hodgkin's disease but there remain many problems to be solved.

### References

BAKEMEIER, R.F., ANDERSON, J.R., COSTELLO, W. & 6 others (1984). BCVPP chemotherapy for advanced Hodgkin's disease: evidence for greater duration of complete remission, greater survival and less toxicity than a MOPP regimen. *Ann. Intern. Med.*, **101**, 447.

BONADONNA, G., SANTORO, A., VALAGUSSA, P. & 4 others (1985). Current status of the Milan trials of Hodgkin's disease in adults. In *Malignant Lymphomas and Hodgkin's Disease*, Cavalli, F., Bonadonna, G. & Rosencweig, M. (eds) p. 299. Martinus Nijhoff: The Hague.

BRITISH NATIONAL LYMPHOMA INVESTIGATION (1986). Randomised study of LOPP against MOPP chemotherapy for advanced Hodgkin's disease. *Radiother. Oncol.*, **7**, 215.

CARBONE, P.P., KAPLAN, H.S., MUSSHOFF, K., SMITHERS, D.W. & TUBIANA, M. (1971). Report of the Committee on Hodgkin's disease staging. *Cancer Res.*, **31**, 1860.

COLMAN, M. & SELBY, P. (1987). Second malignancies and Hodgkin's disease. In *Hodgkin's Disease*, Selby, P. & McElwain, T.J. (eds) p. 361. Blackwell: Oxford.

COX, D.R. (1972). Regression models and life tables. *J. R. Stat. Soc. B*, **34**, 187.

DADY, P.J., MCELWAIN, T.J., AUSTIN, D.E., BARRETT, A. & PECKMAN, M.J. (1982). Five years experience with ChlVPP: effective low-toxicity combination chemotherapy for Hodgkin's disease. *Br. J. Cancer*, **45**, 851.

DE VITA, V.T., SIMON, R.M., HUBBARD, S.M. & 6 others (1980). Curability of advanced Hodgkin's disease with chemotherapy. *Ann. Intern. Med.*, **92**, 587.

KAPLAN, E.L. & MEIER, P. (1978). Non-parametric estimation from incomplete observations. *J. Am. Stat. Assoc.*, **54**, 457.

LONGO, D.L., YOUNG, R.C., WESLEY, M. & 4 others (1986). Twenty years of MOPP therapy for Hodgkin's disease. *J. Clin. Oncol.*, **4**, 1295.

LUKES, R.J. & BUTLER, J.J. (1966). The pathology and nomenclature of Hodgkin's disease. *Cancer Res.*, **26**, 1063.

MCELWAIN, T.J., TOY, J., SMITH, I.E., PECKHAM, M.J. & AUSTIN, D.E. (1977). A combination of chlorambucil, vinblastine, procarbazine, and prednisolone for treatment of Hodgkin's disease. *Br. J. Cancer*, **36**, 276.

MCKENDRICK, J.J., MEAD, G.M., SWEENTENHAM, J. & 4 others (1989). ChlVPP chemotherapy in advanced Hodgkin's disease. *Eur. J. Cancer Clin. Oncol.*, **25**, 557.

PETO, R., PIKE, M.C., ARMITAGE, P. *et al.* (1977). Design and analysis of randomised clinical trials requiring prolonged observation of each patient. *Br. J. Cancer*, **35**, 1.

ROBINSON, B., KINGSTON, J., NOGUERA COSTA, R., MALPAS, J.S., BARRETT, A. & MCELWAIN, T.J. (1984). Chemotherapy and irradiation in childhood Hodgkin's disease. *Arch. Dis. Child.*, **59**, 1162.

RUSSELL, J.A., SELBY, P.J., RUETLER, B.A. & 7 others (1989). Treatment of advanced Hodgkin's disease with high dose melphalan and autologous bone marrow transplantation. *Bone Marrow Transplant.*, **4**, 425.

SELBY, P., MCELWAIN, T.J. & CANELLOS, G. (1987). Chemotherapy of Hodgkin's disease. In *Hodgkin's Disease*, Selby, P. & McElwain, T.J. (eds) p. 269. Blackwell: Oxford.

SUTCLIFFE, S.B. (1987). Infertility and gonadal function in Hodgkin's disease. In *Hodgkin's Disease*, Selby, P. & McElwain, T.J (eds) p. 339. Blackwell: Oxford.

TUCKER, M.A., COLEMAN, C.N., COX, R.S., VARGHESE, A. & ROSENBERG, S.A. (1988). Risk of second cancers after treatment for Hodgkin's disease. *N. Engl. J. Med.*, **318**, 76.

WAGSTAFF, J., GREGORY, W.M., SWINDELL, R., CROWTHER, D. & LISTER, T.A. (1988). Prognostic factors for survival in stage IIIB and IV Hodgkin's disease: a multivariate analysis comparing two specialist treatment centres. *Br. J. Cancer*, **58**, 487.

WORLD HEALTH ORGANIZATION (1979). *WHO Handbook for Reporting Results of Cancer Treatment*. WHO: Geneva.

ZULIAN, G.B., SELBY, P., MILAN, S. & 5 others (1989). High dose melphalan, BCNU, and etoposide with autologous bone marrow transplantation for Hodgkin's disease. *Br. J. Cancer*, **59**, 631.


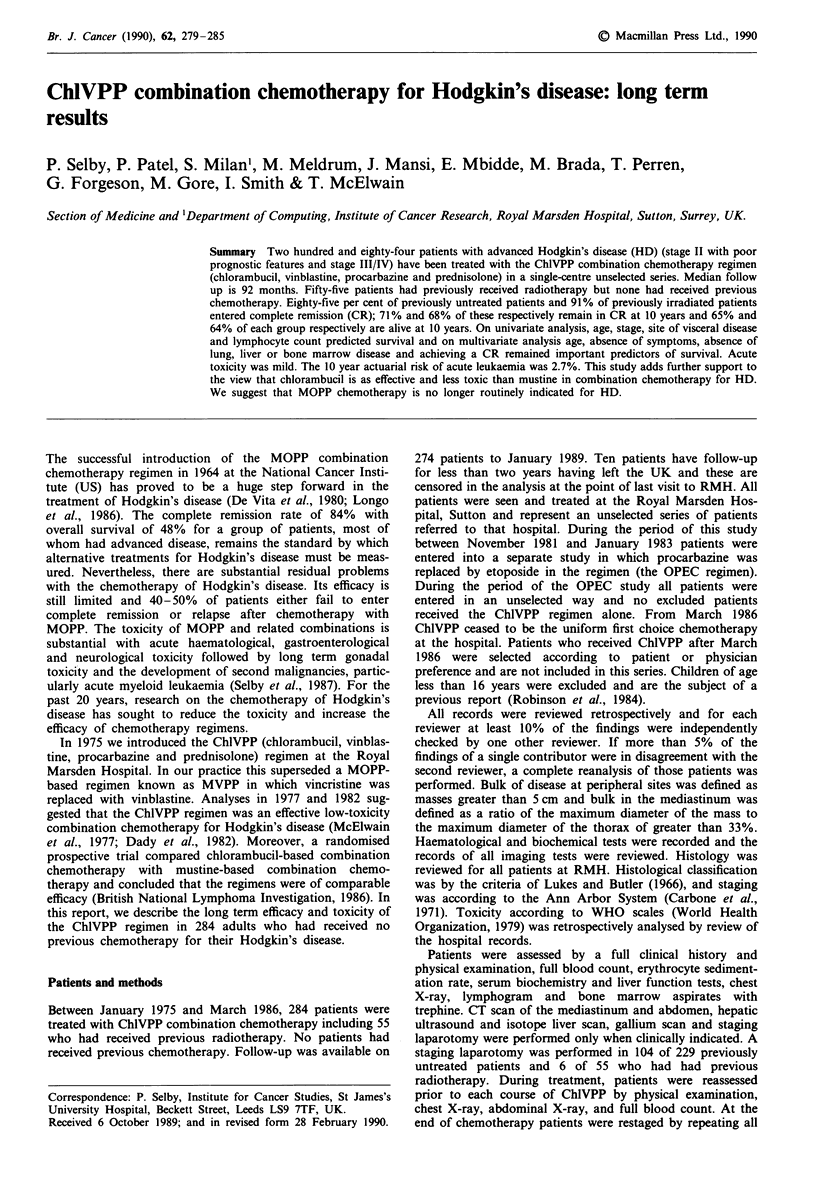

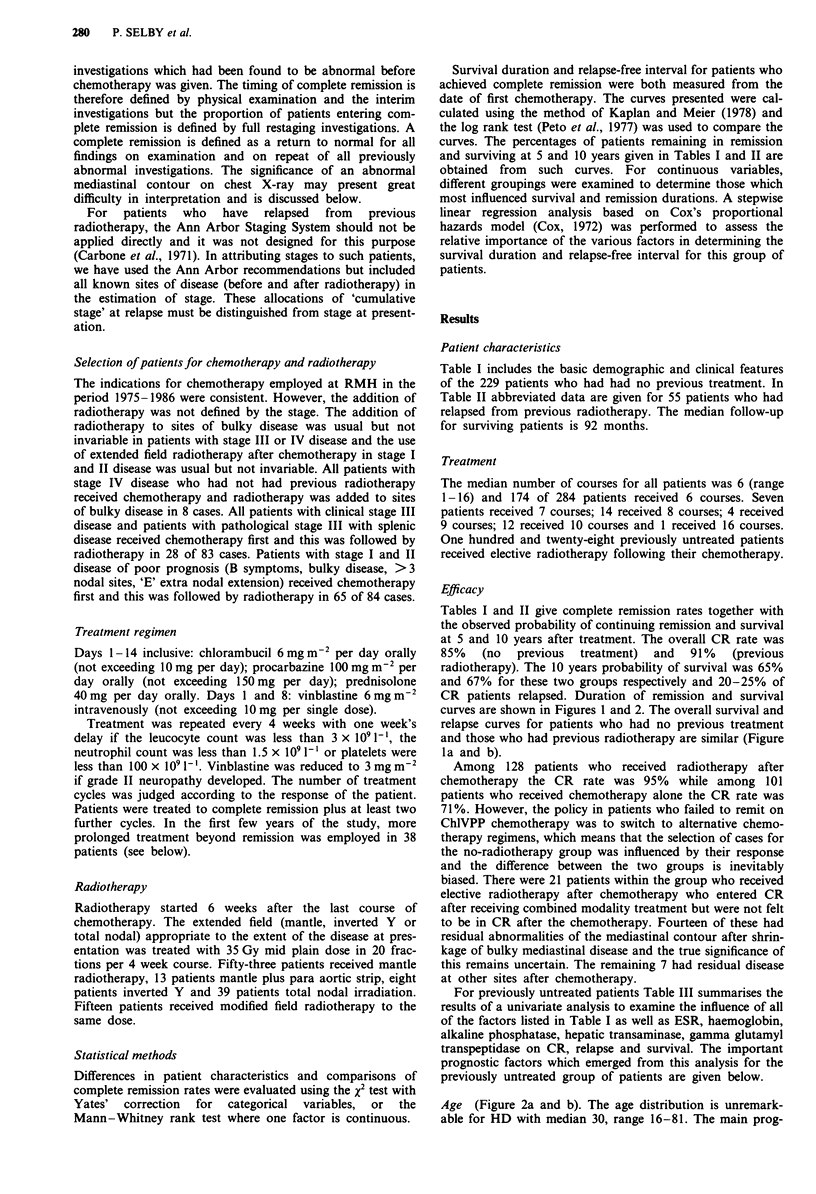

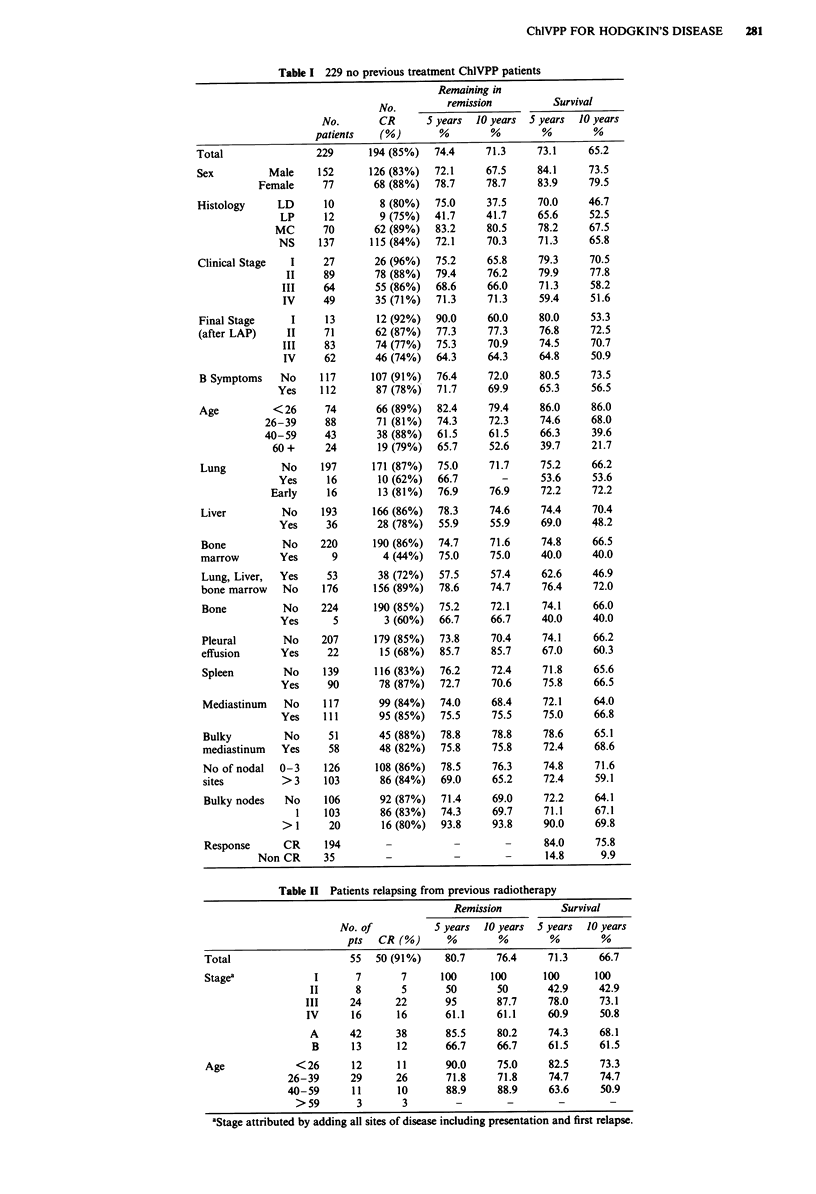

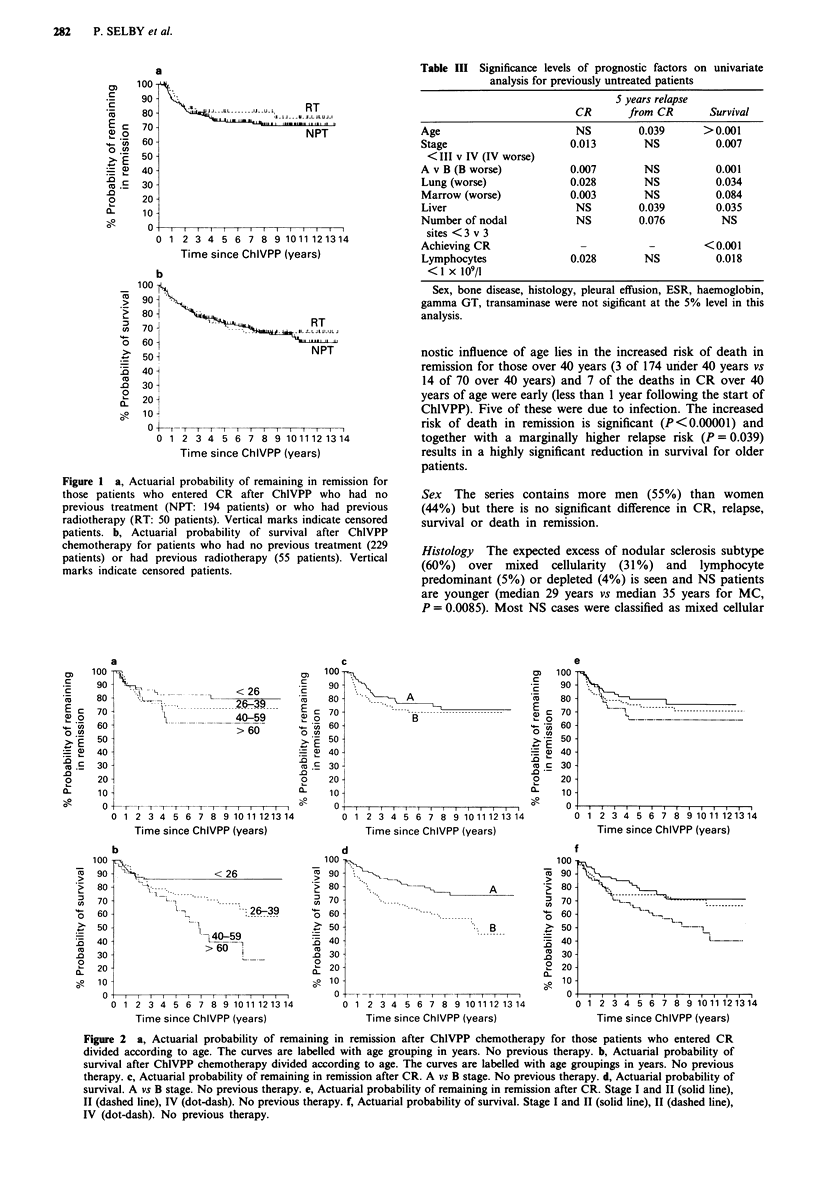

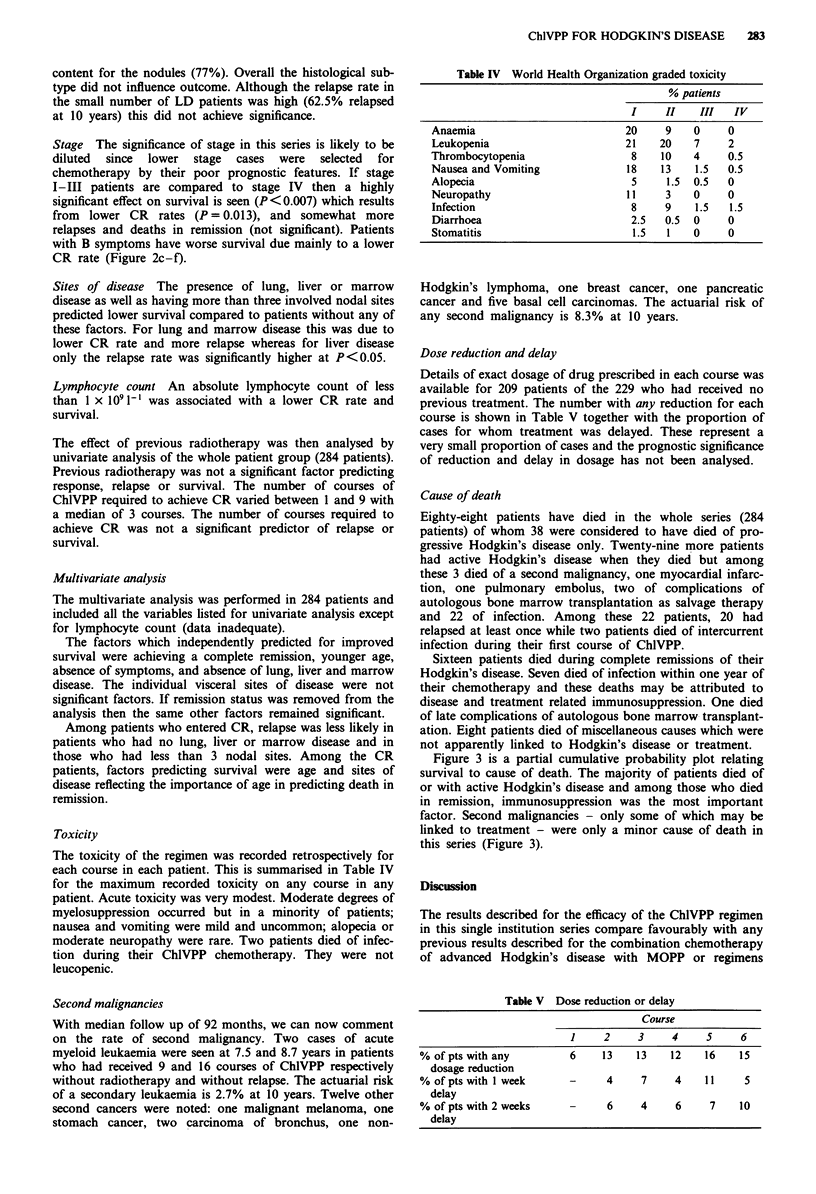

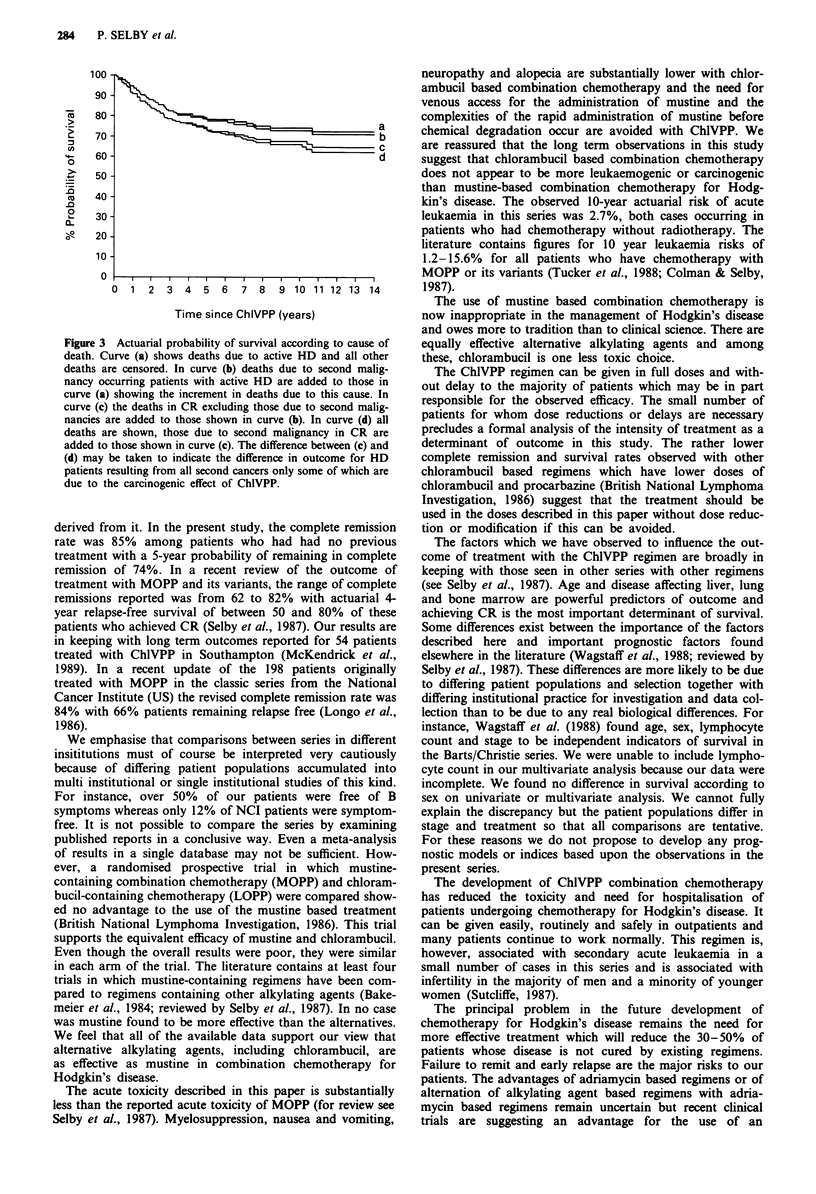

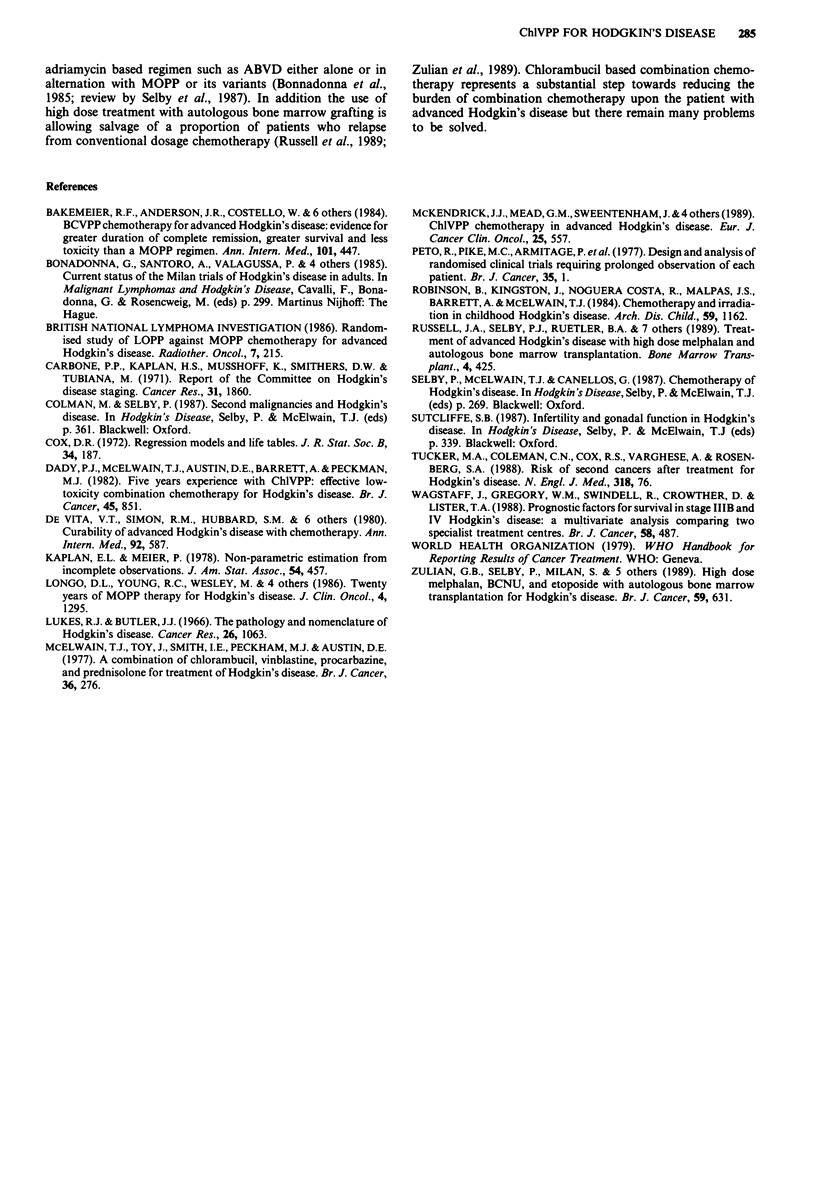


## References

[OCR_01275] Bakemeier R. F., Anderson J. R., Costello W., Rosner G., Horton J., Glick J. H., Hines J. D., Berard C. W., DeVita V. T. (1984). BCVPP chemotherapy for advanced Hodgkin's disease: evidence for greater duration of complete remission, greater survival, and less toxicity than with a MOPP regimen. Results of the Eastern Cooperative Oncology Group study.. Ann Intern Med.

[OCR_01291] Carbone P. P., Kaplan H. S., Musshoff K., Smithers D. W., Tubiana M. (1971). Report of the Committee on Hodgkin's Disease Staging Classification.. Cancer Res.

[OCR_01305] Dady P. J., McElwain T. J., Austin D. E., Barrett A., Peckham M. J. (1982). Five years' experience with ChlVPP: effective low-toxicity combination chemotherapy for Hodgkin's disease.. Br J Cancer.

[OCR_01311] DeVita V. T., Simon R. M., Hubbard S. M., Young R. C., Berard C. W., Moxley J. H., Frei E., Carbone P. P., Canellos G. P. (1980). Curability of advanced Hodgkin's disease with chemotherapy. Long-term follow-up of MOPP-treated patients at the National Cancer Institute.. Ann Intern Med.

[OCR_01320] Longo D. L., Young R. C., Wesley M., Hubbard S. M., Duffey P. L., Jaffe E. S., DeVita V. T. (1986). Twenty years of MOPP therapy for Hodgkin's disease.. J Clin Oncol.

[OCR_01325] Lukes R. J., Butler J. J. (1966). The pathology and nomenclature of Hodgkin's disease.. Cancer Res.

[OCR_01329] McElwain T. J., Toy J., Smith E., Peckham M. J., Austin D. E. (1977). A combination of chlorambucil, vinblastine, procarbazine and prednisolone for treatment of Hodgkin's disease.. Br J Cancer.

[OCR_01335] McKendrick J. J., Mead G. M., Sweetenham J., Jones D. H., Williams C. J., Ryall R., Whitehouse J. M. (1989). ChlVPP chemotherapy in advanced Hodgkin's disease.. Eur J Cancer Clin Oncol.

[OCR_01340] Peto R., Pike M. C., Armitage P., Breslow N. E., Cox D. R., Howard S. V., Mantel N., McPherson K., Peto J., Smith P. G. (1977). Design and analysis of randomized clinical trials requiring prolonged observation of each patient. II. analysis and examples.. Br J Cancer.

[OCR_01347] Robinson B., Kingston J., Nogueira Costa R., Malpas J. S., Barrett A., McElwain T. J. (1984). Chemotherapy and irradiation in childhood Hodgkin's disease.. Arch Dis Child.

[OCR_01350] Russell J. A., Selby P. J., Ruether B. A., Mbidde E. K., Ashley S., Zulian G., Berry J., Houwen B., Jones A. R., Poon M. C. (1989). Treatment of advanced Hodgkin's disease with high dose melphalan and autologous bone marrow transplantation.. Bone Marrow Transplant.

[OCR_01368] Tucker M. A., Coleman C. N., Cox R. S., Varghese A., Rosenberg S. A. (1988). Risk of second cancers after treatment for Hodgkin's disease.. N Engl J Med.

[OCR_01371] Wagstaff J., Gregory W. M., Swindell R., Crowther D., Lister T. A. (1988). Prognostic factors for survival in stage IIIB and IV Hodgkin's disease: a multivariate analysis comparing two specialist treatment centres.. Br J Cancer.

[OCR_01381] Zulian G. B., Selby P., Milan S., Nandi A., Gore M., Forgeson G., Perren T. J., McElwain T. J. (1989). High dose melphalan, BCNU and etoposide with autologous bone marrow transplantation for Hodgkin's disease.. Br J Cancer.

